# Supplementary material for: Cell state-dependent allelic effects and contextual Mendelian randomization analysis for human brain phenotypes
Source: Nat Genet. 2025 Jan 10;57(2):358–68. doi: 10.1038/s41588-024-02050-9 (PMC11821528; doi:10.1038/s41588-024-02050-9)
Supplement: Supplementary file 1 — Supplementary Figs. 1–16. [file 41588_2024_2050_MOESM1_ESM.pdf]

nature genetics

Supplementary information https://doi.org/10.1038/s41588-024-02050-9

Cell state-dependent allelic effects and contextual Mendelian randomization analysis for human brain phenotypes

In the format provided by the authors and unedited

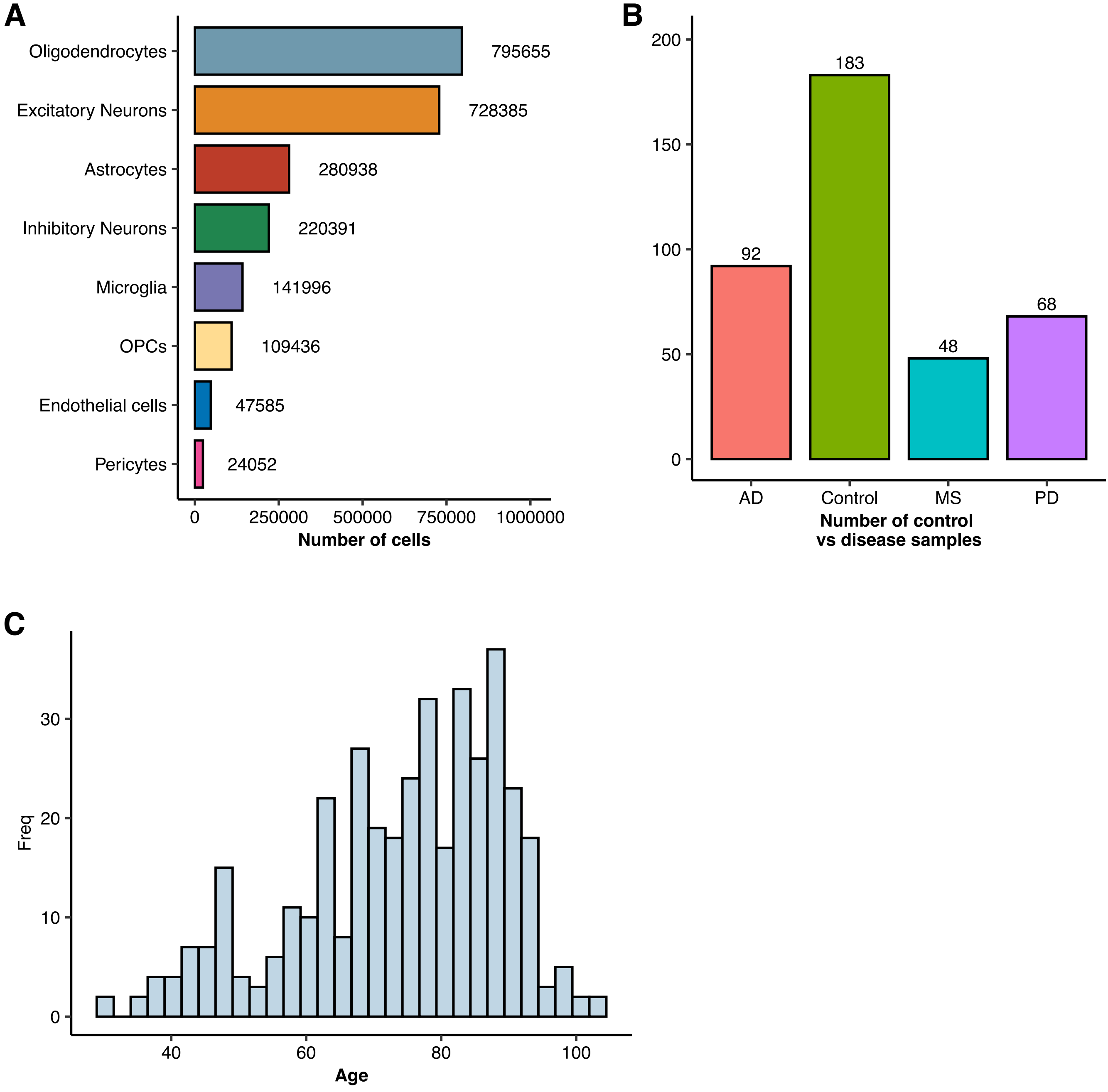

Supplementary Figure 1 - Dataset overview. A, Overview of cell-type distribution across full (n=391) dataset. In total, we retained ~2.35 million cells. **B,** Distribution of cases versus control samples in dataset. **C,** Distribution of patient age at death.

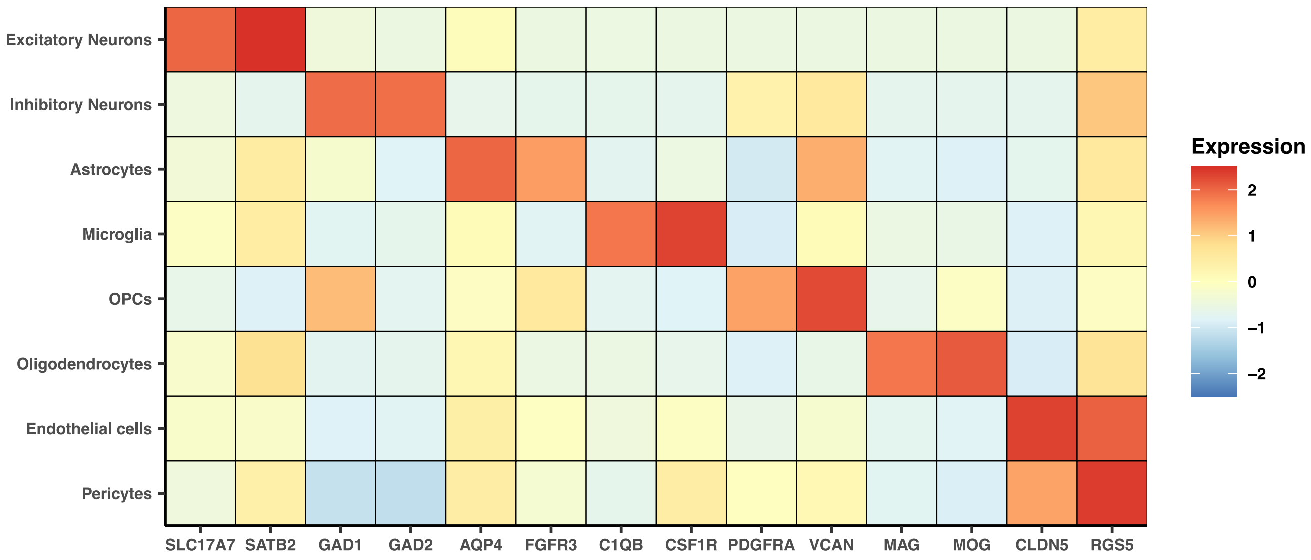

Supplementary Figure 2 - Mean marker gene expression per cell-type. The scaled mean expression of pseudobulked expression matrices (log transformed counts per million, CPM) is shown for each cell-type.

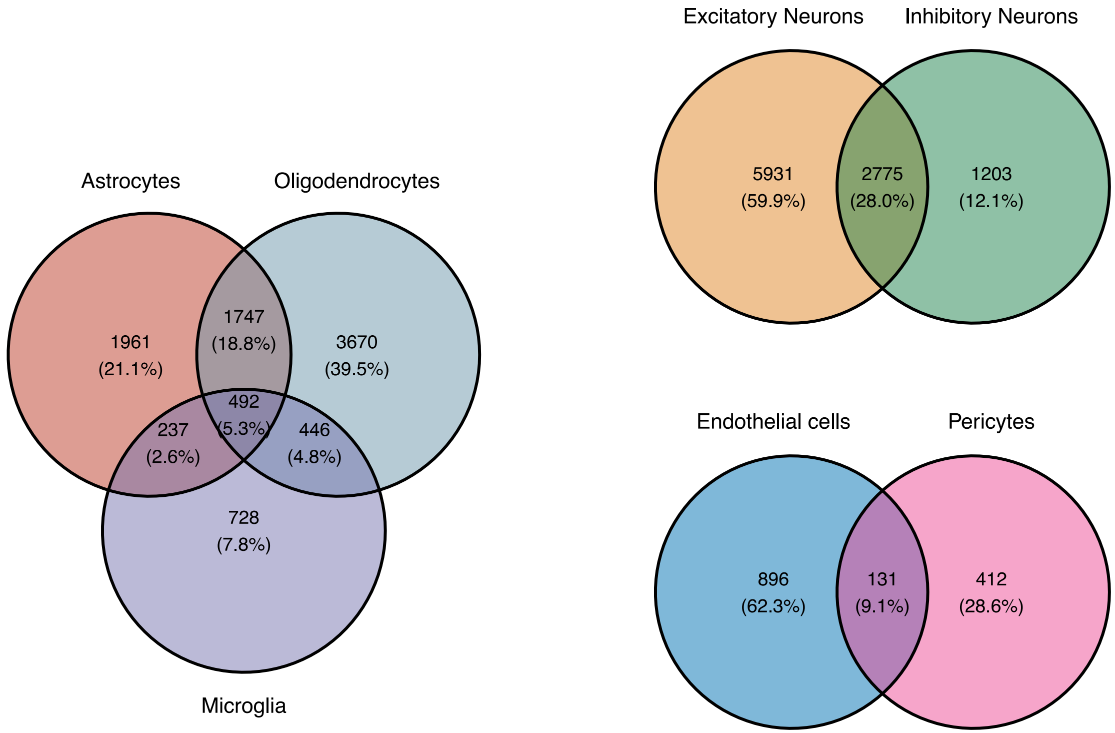

Supplementary Figure 3 - Common eGenes between cell-types. Each number represents the shared number of eGenes (intersection) and the percentage it represents of the total number of eGenes between sets. For example, there were 2,775 eGenes found to be shared between Excitatory Neurons and Inhibitory Neurons, or 28.0% of the total set (9,909 unique eGenes).

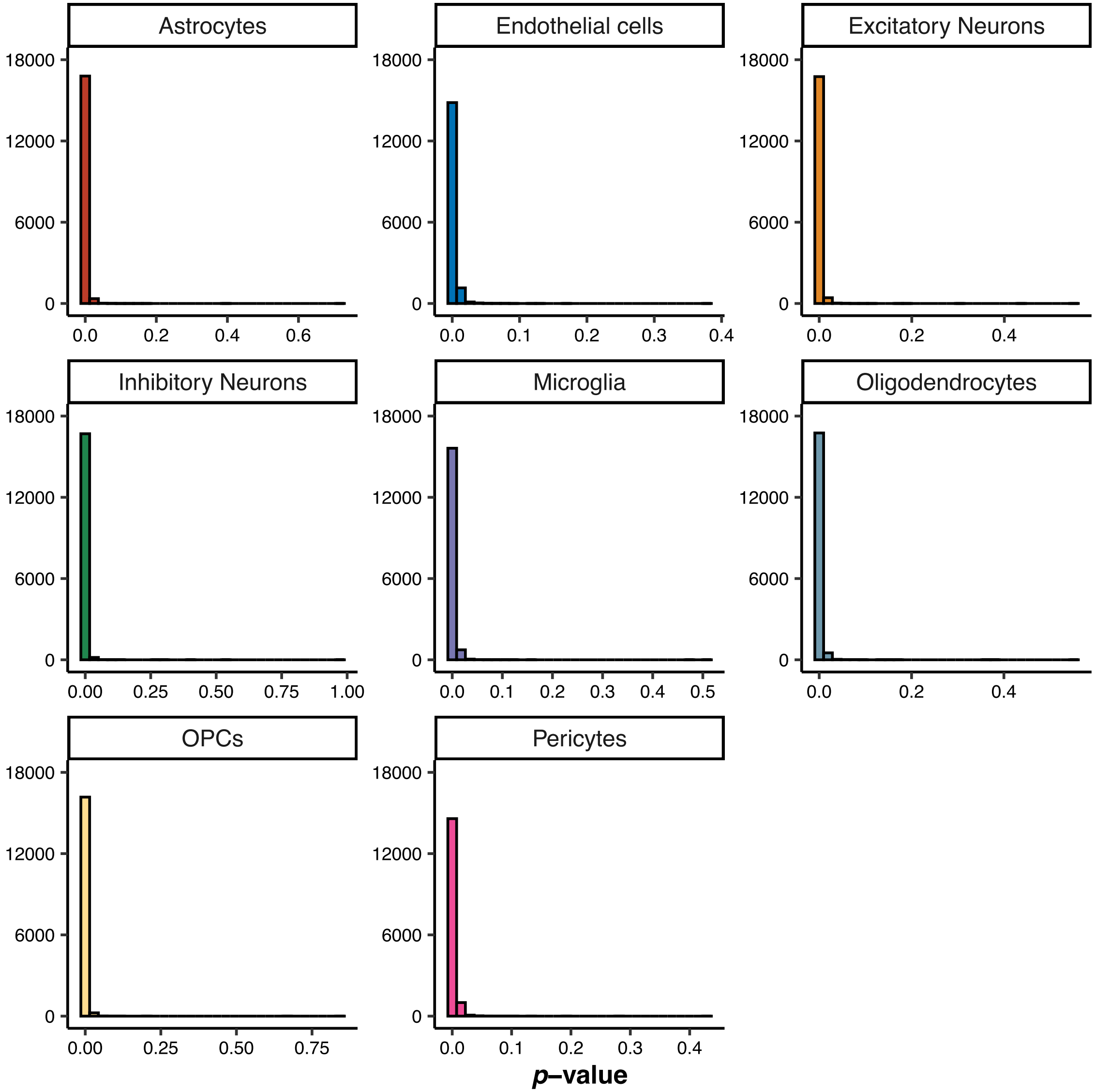

Supplementary Figure 4 - eQTL *p*-value distributions. We obtained the reported *p*-values from MatrixEQTL, which fits a linear model between SNP dosage and gene expression (including covariates) and retained the top associated SNP for each gene. The x-axis represents the *p*-value and the y-axis represents the number of SNP-gene associations in each bin (bin size = 30).

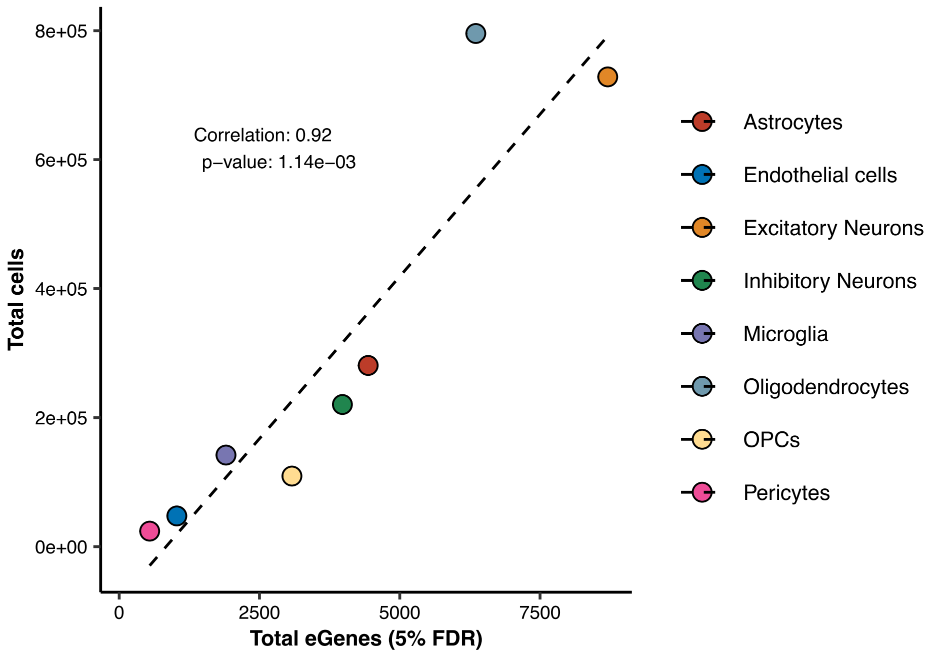

Supplementary Figure 5 - Impact of cell numbers on eQTL discovery. The x-axis represents the total eGenes discovered at 5% FDR (as estimated by MatrixEQTL on all *p*-values obtained from the linear models) versus total cells for that cell-type (y-axis). The correlation coefficient was estimated using Pearson correlation test ($r = 0.92$, p-value = 1.14×10^{-3}) between total cell numbers and eGenes across the cell-types.

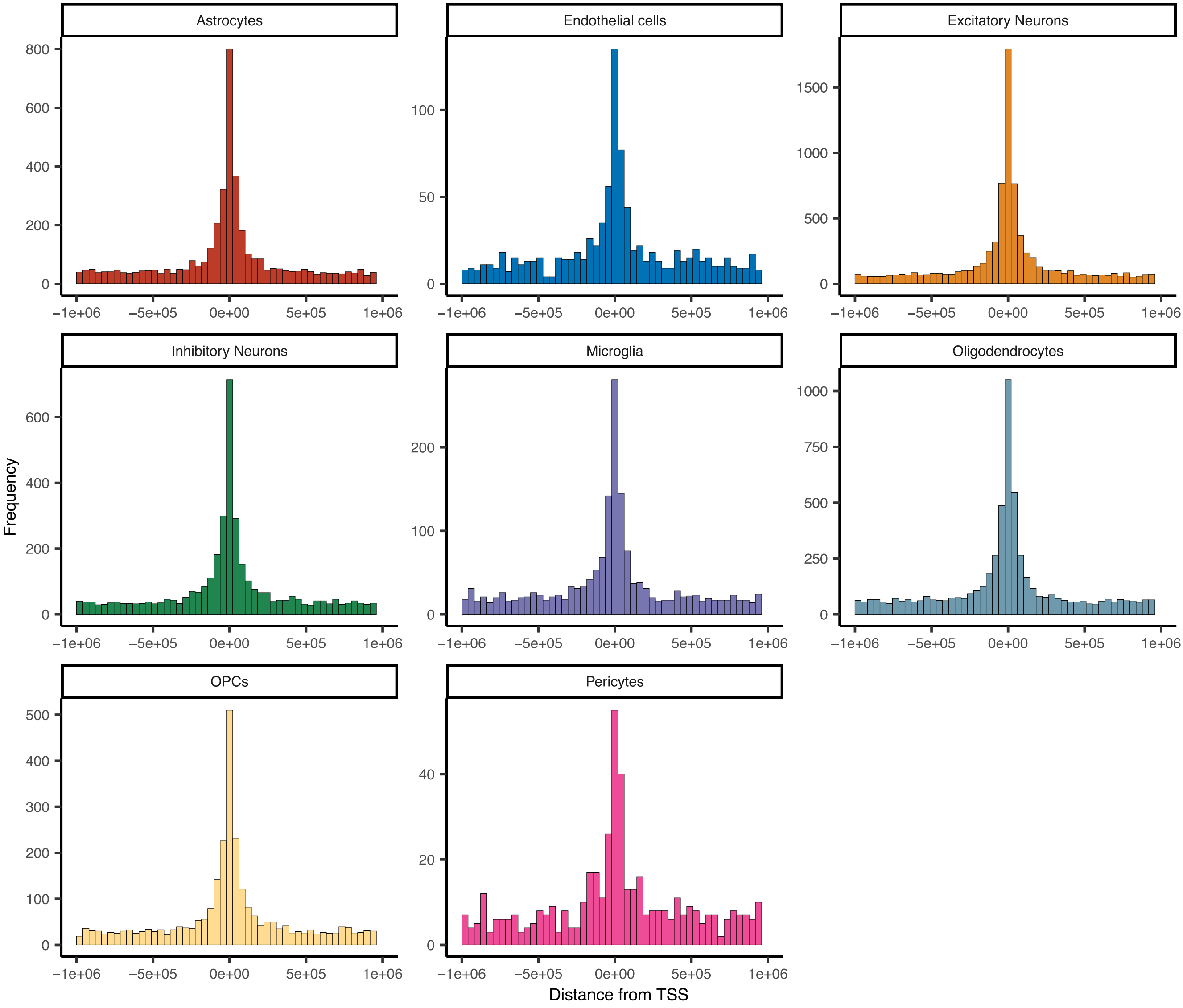

Supplementary Figure 6 - eSNP distance to TSS. Distance to transcription start site (TSS) was calculated based on known transcription start sites (Ensembl) for each gene tested, and the top regulatory SNP for each eGene. The x-axis represents distance from the start site (positive indicates downstream, negative indicates upstream). The y-axis represents the frequency in each bin (bin size = 50).

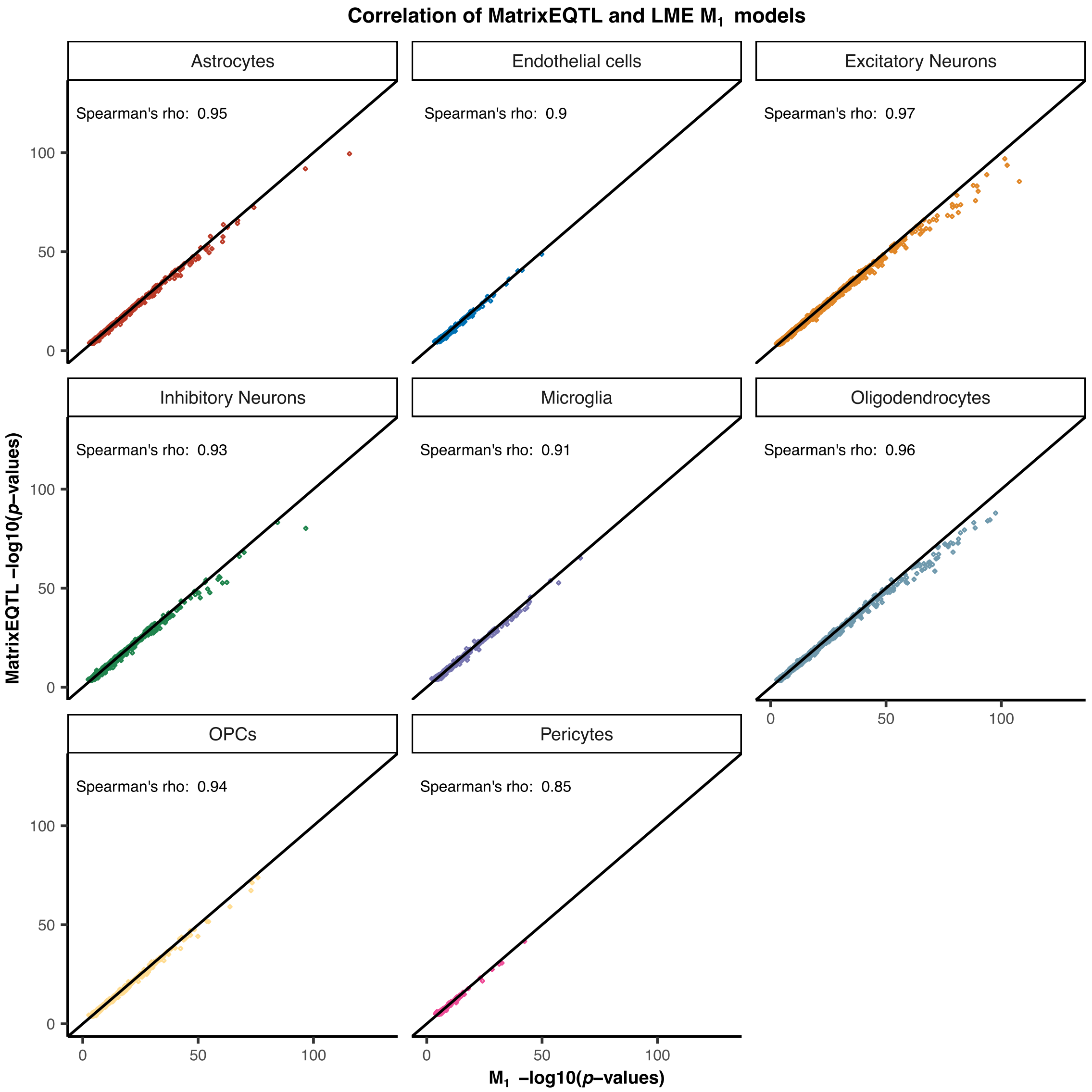

Supplementary Figure 7 - Comparison of M_1 and MatrixEQTL *p*-values using Spearman Rank Correlation. We remodelled significant associations between the top SNP and each eGene (< 5% FDR) using mixed effects models including a nested random effect on disease status and sample source. The y-axis represents the -log_{10}(*p*-values) obtained from the linear models implemented in MatrixEQTL, whereas the x-axis represents the -log_{10}(*p*-values) obtained from linear mixed effecs models (M_1). The correlation metric was obtained from a Spearman's Rank Correlation test between the two sets of *p*-values for each cell-type.

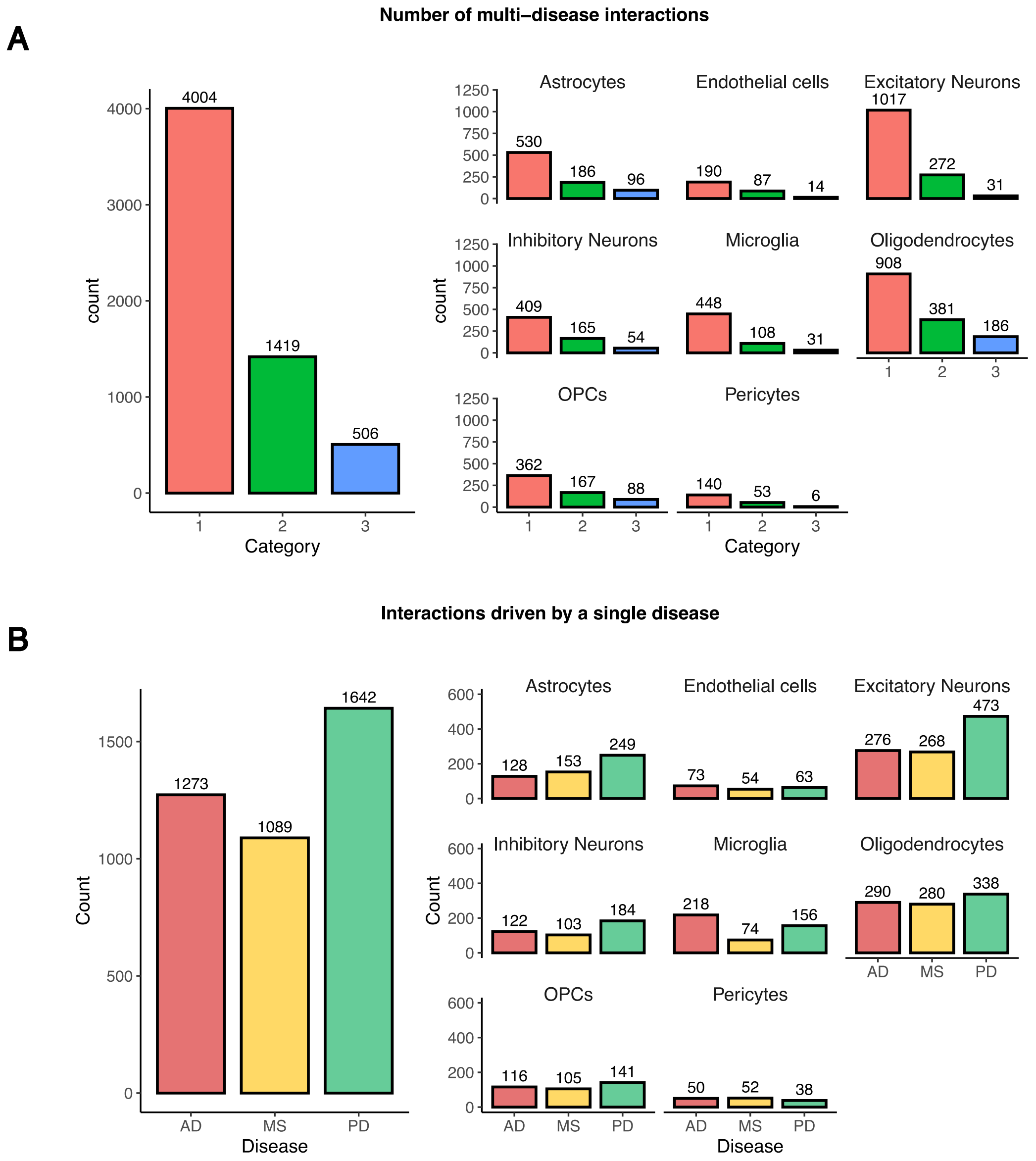

Supplementary Figure 8 - Interaction of disease with eQTL associations. **A**, number of interaction eQTLs (interaction *q*-value < 0.05) affected by one (red), two (green) or three (blue) disease categories for the full set of eQTLs (left) and divided by cell-type (right). **B**, Number of interaction eQTLs affected by a single disease, separated by disease category for overall (left) and by cell-type (right).

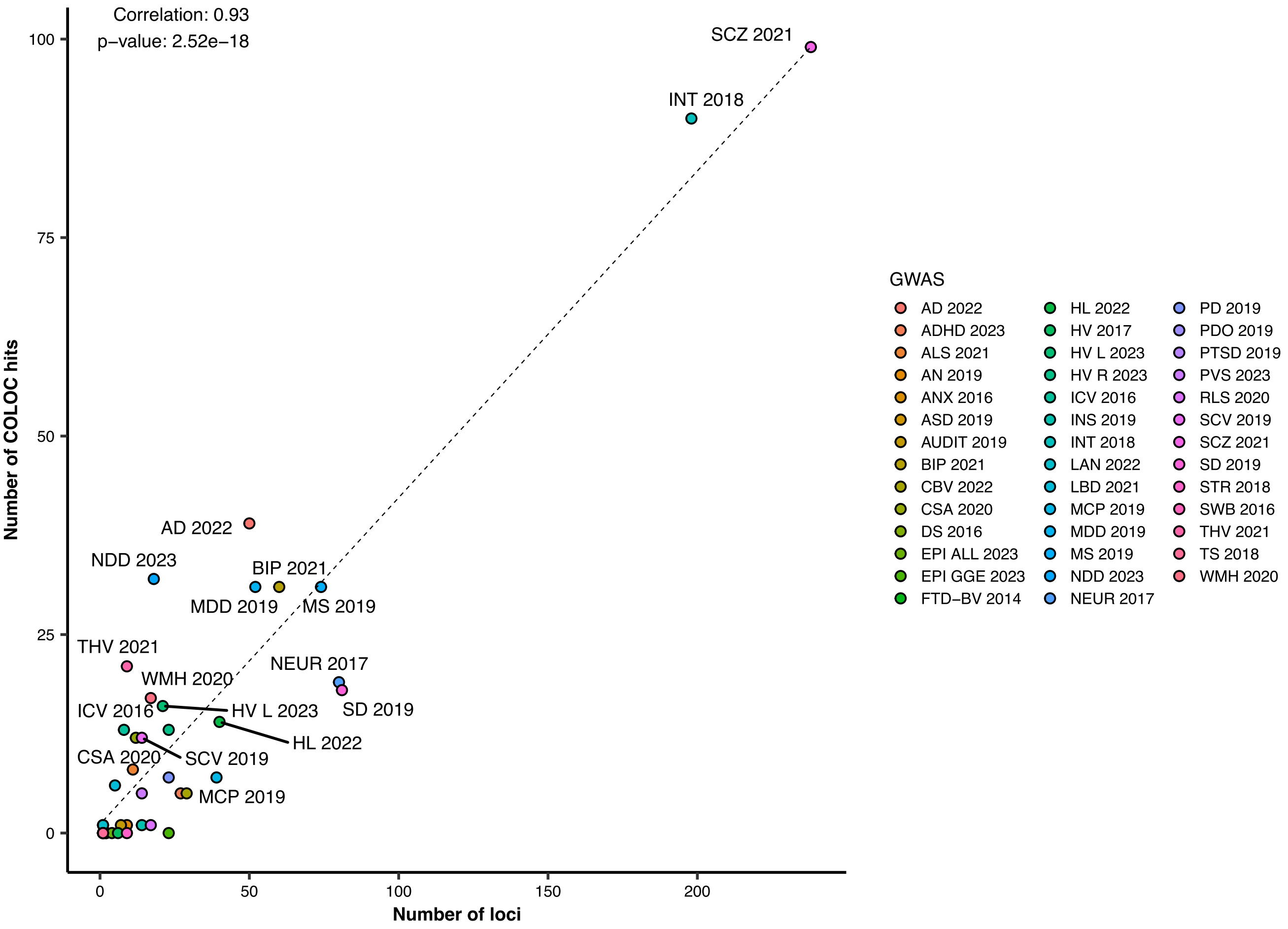

Supplementary Figure 9 - Number of COLOC hits discovered per trait using snRNA-seq brain eQTLs (n=391). The total number of COLOC hits (y-axis) that can be discovered is highly dependent on the number of genomic loci discovered at genome-wide significance (p-value $< 5 \times 10^{-8}$, x-axis) based on the observed pearson correlation coefficient ($r = 0.93$, p-value $= 2.52 \times 10^{-18}$).

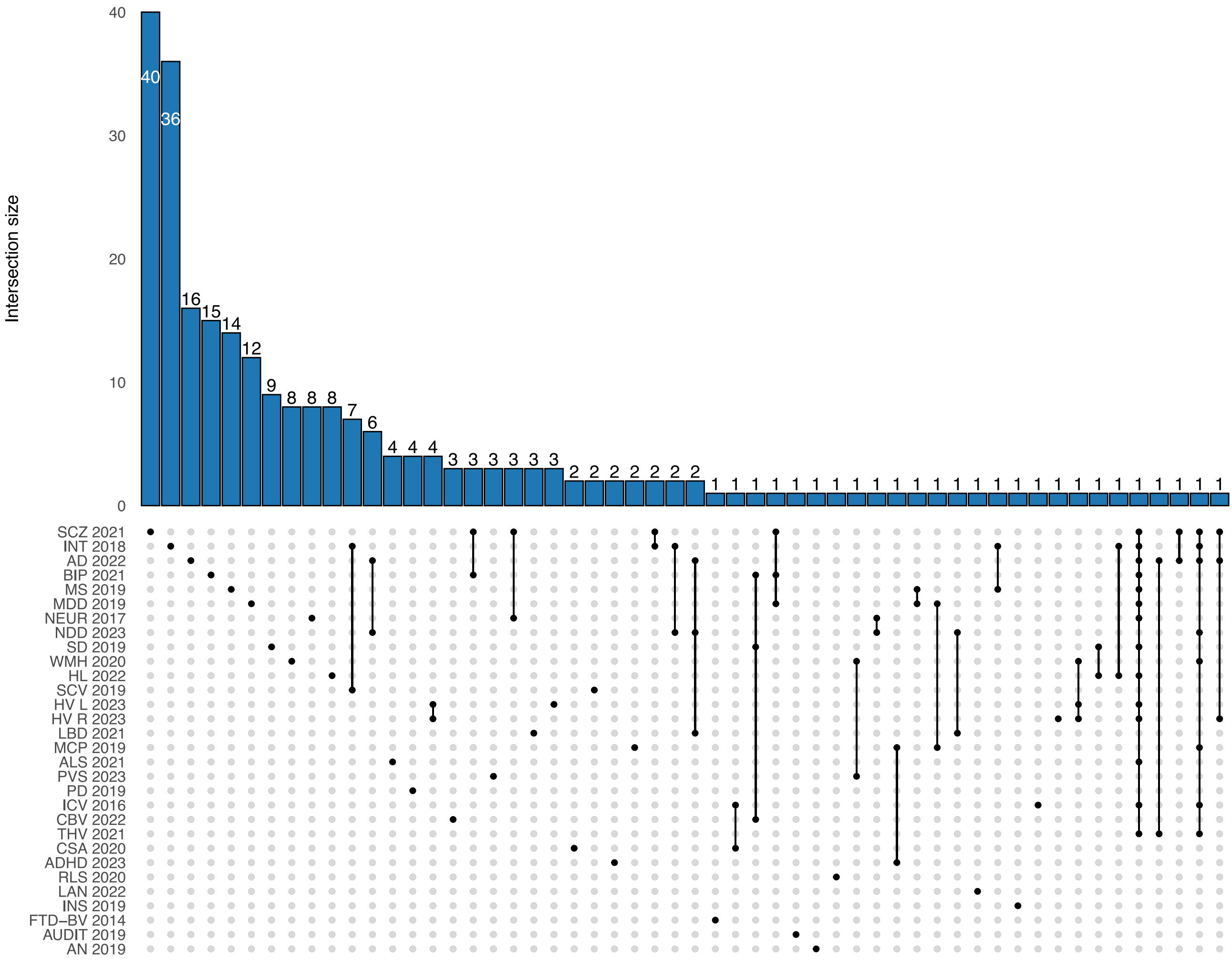

Supplementary Figure 10 - Intersecting genes for colocalisations across 41 traits. We performed genetic colocalisation using single-cell eQTL data with 41 neurological, psychiatric, behavioural and structural traits and retained colocalisations with a posterior probability (PP.H4) > 0.8 . The numbers above each bar represent the number of unique genes in that set (e.g 40 genes were found to colocalise with one or more cell-types in schizophrenia (SCZ), 7 genes were shared between intelligence (INT) and subcortical volume (SCV) in one or more cell-types).

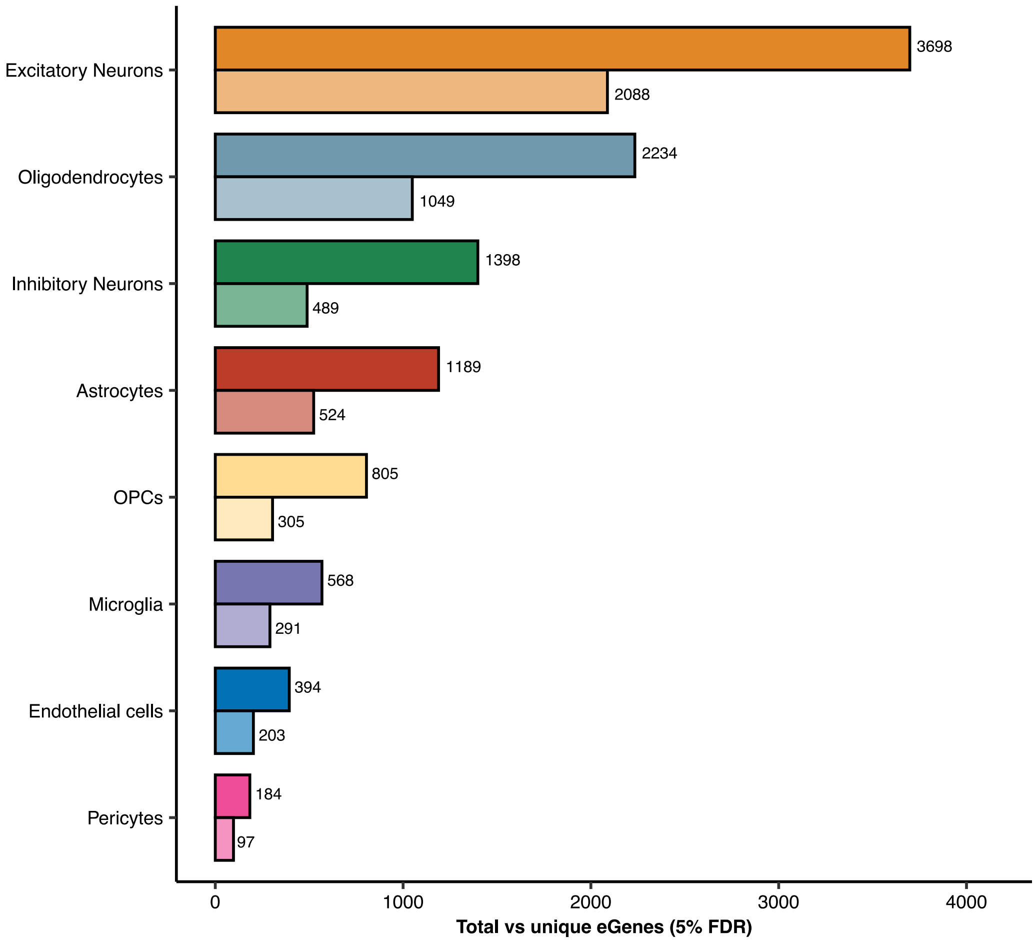

Supplementary Figure 11 - Overview of eQTL discovery in a subset cohort consisting exclusively of control samples (n=183). Each bar represents the number of genes regulated by at least one SNP (FDR < 5%) per cell-type. Darker coloured bars represents total genes, lighter coloured bars represents the number of genes unique to that cell-type.

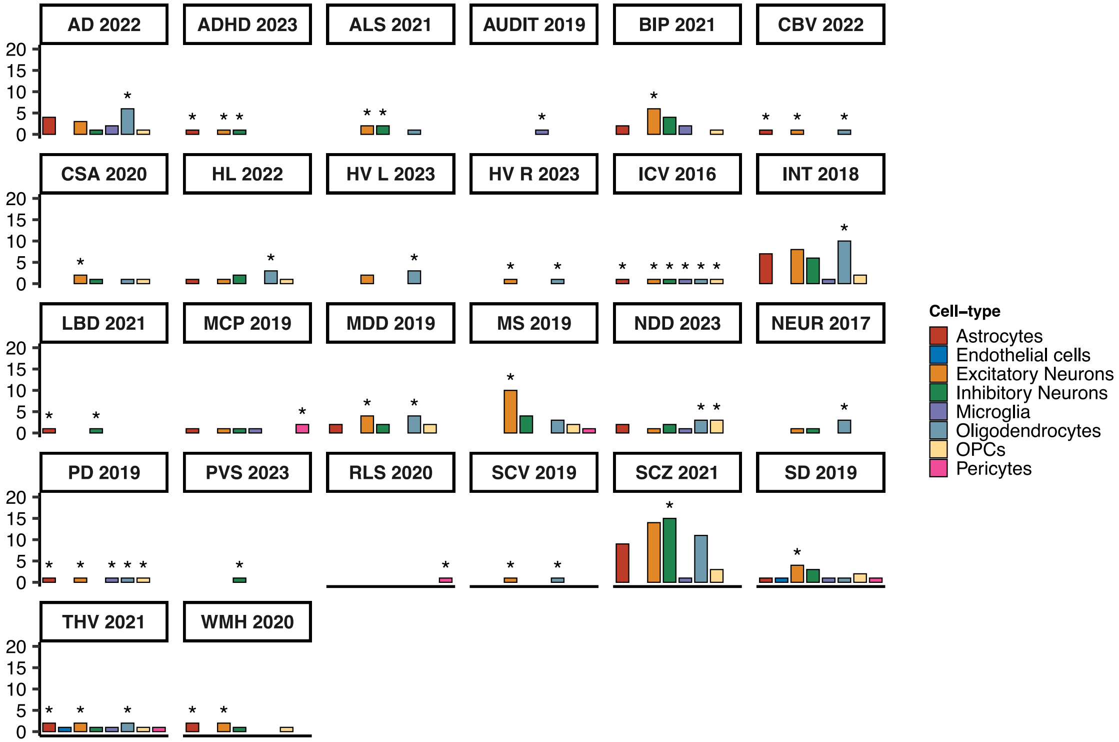

Supplementary Figure 12 - Overview of number of colocalisation across cell-types and neurological phenotypes, using eQTLs discovered in the control only (n=183) cohort. Each colocalisation represents a cell-type x gene x trait triplet, filtered to colocalisations with a posterior probability (PP.H4) above 0.8. Stars (*) indicate the cell-type with the most colocalisations in a single trait.

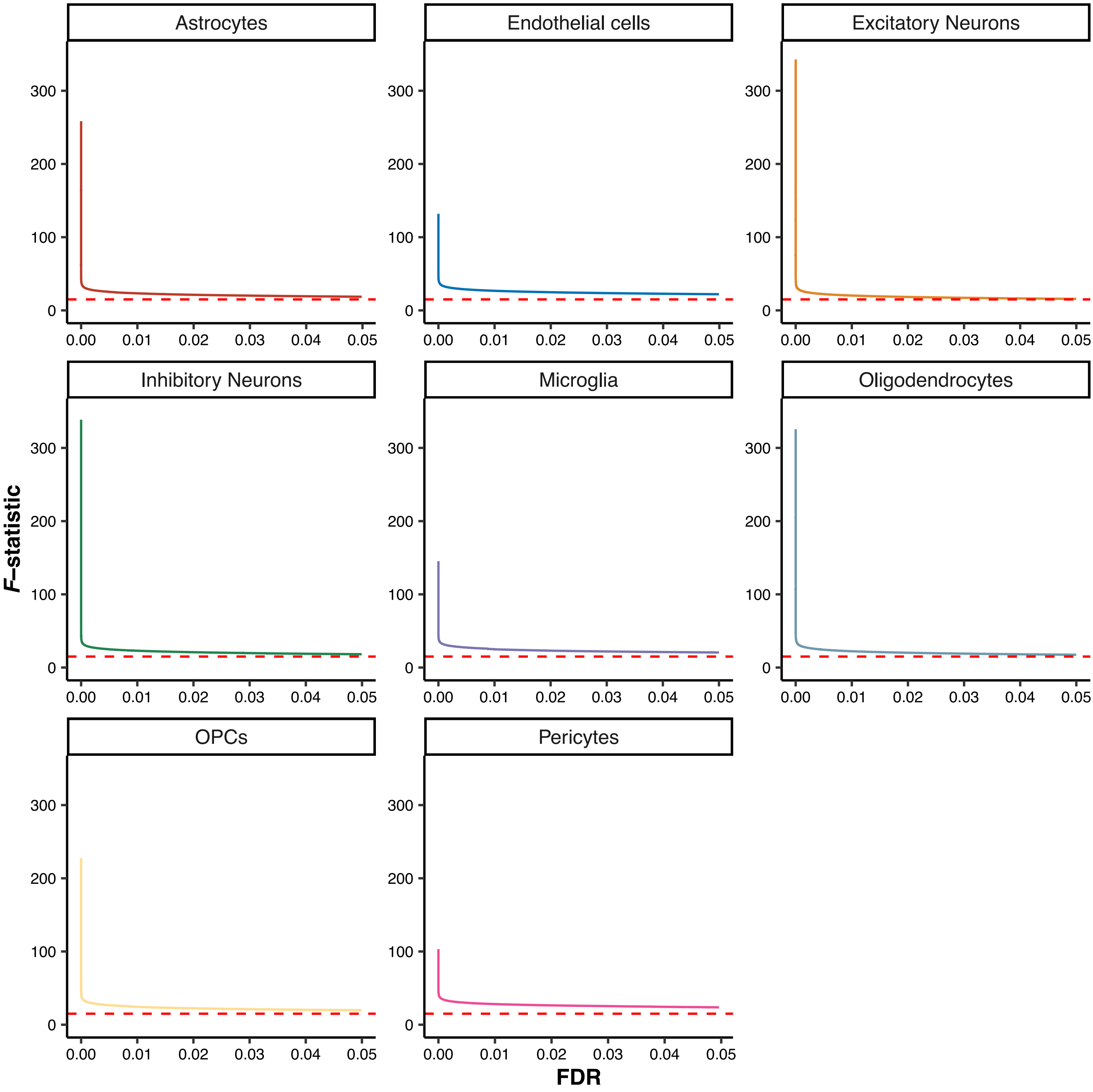

Supplementary Figure 13 - *F*-statistic distribution of genetic instrumental variables (IVs) for MR. For each cell-type, the F-statistic is shown (y-axis) against the corresponding FDR (x-axis) across all eQTLs (FDR < 0.05). The red dotted line represents an *F*-statistic of 15. The y-axis scale is limited to *F*-statistic=350 for comparison with less abundant cell-types**.**

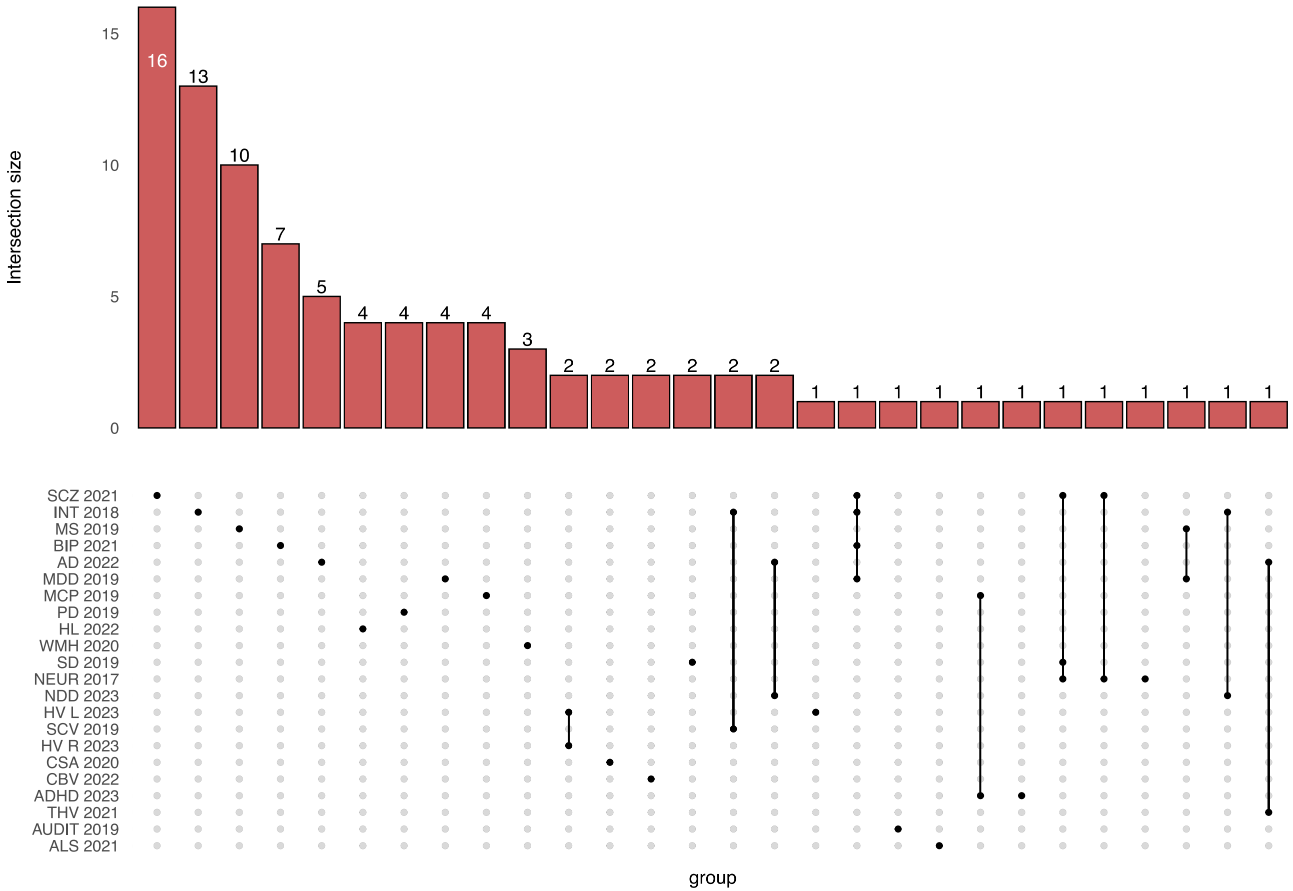

Supplementary Figure 14 - Intersecting genes for significant MR associations. We performed Mendelian Randomisation (MR) using single-cell eQTL data on a cohort of control-only samples (n=183), retaining significant associations determined by Inverse Variance Weighting (IVW) fixed effects modelling (*p*-value < 0.05). Each number above the bars represent the number of unique genes in that set for one or more cell-types. For example, we found 16 genes unique to schizophrenia (SCZ), and 2 were shared between intelligence (INT) and subcortical volume (SCV). In total, there were 94 unique genes across the whole dataset in at least one trait and one cell-type (140 significant gene / cell-type / trait triplets in total, excluding *MAPT/HLA* regions).

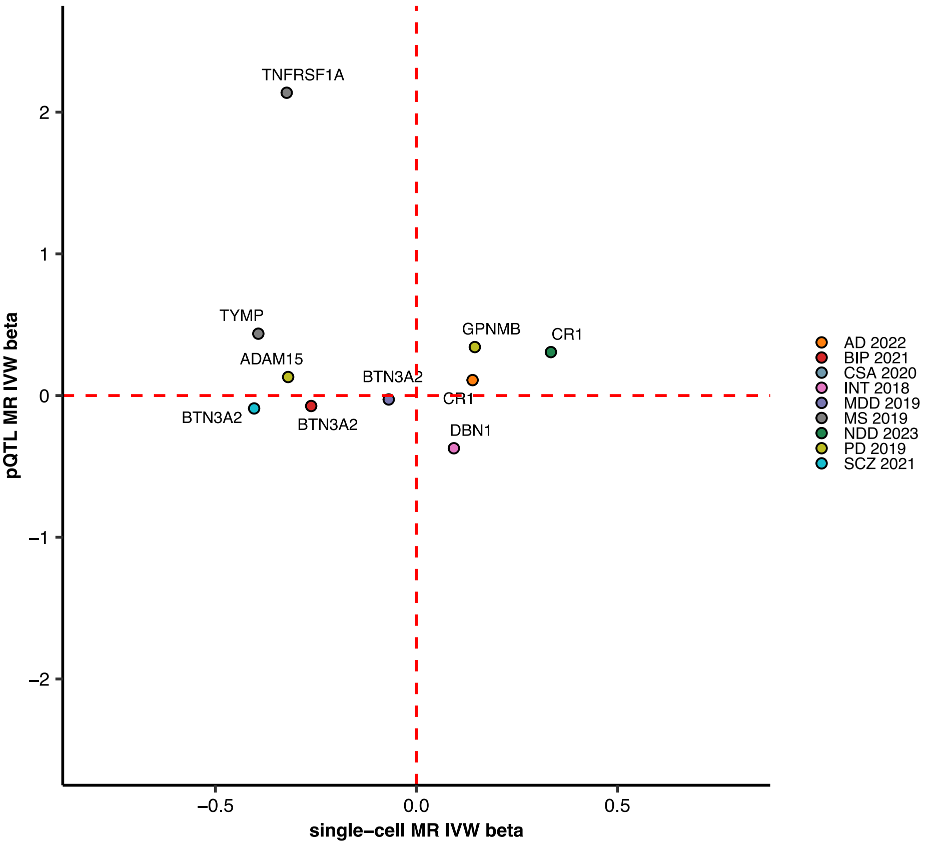

Supplementary Figure 15 - MR effect sizes of pQTLs (UKB-PPP) and neurological traits. We conducted Mendelian Randomisation using published pQTL summary statistics from the UKB-PPP, using genetic variants significantly associated with changes in protein abundance. The effect sizes represent the Inverse-Weighted Variance (IVW) MR estimate for the pQTL tests (y-axis) against the IVW estimates from our cell-type specific MR inferences (x-axis).

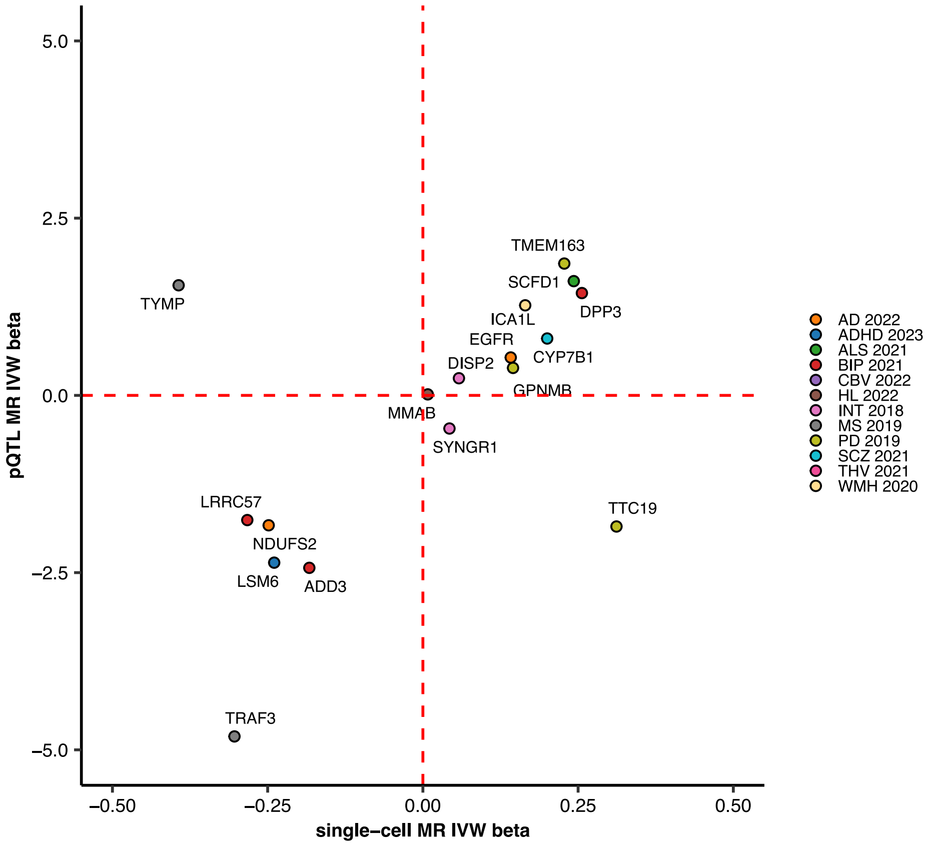

Supplementary Figure 16 - MR effect sizes of pQTLs (Robins et al.,) and neurological traits. We conducted Mendelian Randomisation using published pQTL summary statistics (Robins et al.,), using genetic variants significantly associated with changes in protein abundance. The effect sizes represent the Inverse-Weighted Variance (IVW) MR estimate for the pQTL tests (y-axis) against the IVW estimates from our cell-type specific MR inferences (x-axis).
