# Supplementary material for: Cell state-dependent allelic effects and contextual Mendelian randomization analysis for human brain phenotypes
Source: Nat Genet. 2025 Jan 10;57(2):358–68. doi: 10.1038/s41588-024-02050-9 (PMC11821528; doi:10.1038/s41588-024-02050-9)
Supplement: Supplementary file 2 — Reporting Summary [file 41588_2024_2050_MOESM2_ESM.pdf]

nature portfolio

Corresponding author(s): Michael Johnson

Last updated by author(s): Nov 7, 2024

# Reporting Summary

Nature Portfolio wishes to improve the reproducibility of the work that we publish. This form provides structure for consistency and transparency in reporting. For further information on Nature Portfolio policies, see our Editorial Policies and the Editorial Policy Checklist.

## Statistics

For all statistical analyses, confirm that the following items are present in the figure legend, table legend, main text, or Methods section.

| n/a | Confirmed | |
|---|---|---|
| ☐ | ☒ | The exact sample size (*n*) for each experimental group/condition, given as a discrete number and unit of measurement |
| ☐ | ☒ | A statement on whether measurements were taken from distinct samples or whether the same sample was measured repeatedly |
| ☐ | ☒ | The statistical test(s) used AND whether they are one- or two-sided<br>*Only common tests should be described solely by name; describe more complex techniques in the Methods section.* |
| ☐ | ☒ | A description of all covariates tested |
| ☐ | ☒ | A description of any assumptions or corrections, such as tests of normality and adjustment for multiple comparisons |
| ☐ | ☒ | A full description of the statistical parameters including central tendency (e.g. means) or other basic estimates (e.g. regression coefficient) AND variation (e.g. standard deviation) or associated estimates of uncertainty (e.g. confidence intervals) |
| ☐ | ☒ | For null hypothesis testing, the test statistic (e.g. *F*, *t*, *r*) with confidence intervals, effect sizes, degrees of freedom and *P* value noted<br>*Give P values as exact values whenever suitable.* |
| ☐ | ☒ | For Bayesian analysis, information on the choice of priors and Markov chain Monte Carlo settings |
| ☐ | ☒ | For hierarchical and complex designs, identification of the appropriate level for tests and full reporting of outcomes |
| ☐ | ☒ | Estimates of effect sizes (e.g. Cohen's *d*, Pearson's *r*), indicating how they were calculated |

*Our web collection on statistics for biologists contains articles on many of the points above.*

## Software and code

Policy information about availability of computer code

| | |
|---|---|
| Data collection | No software was used for data collection. |
| Data analysis | Packages or software used in this manuscript include the following; Michigan Imputation Server (version 1.6.3), Eagle (2.4), plink (2.0), bcftools (1.18), Cellranger (5.0.1), Cellranger-ARC (2.0.2), DropletUtils (1.22), Seurat (v4), DoubletFinder (2.0), edgeR (3.4.2), MatrixEQTL (2.3), lmerTest (3.1),qvalue (2.34),MungeSumstats (1.10.1),ieugwasr (1.0.1), coloc (5.2.3), MendelianRandomization (0.10).<br><br>Scripts used for data analysis are available here. https://github.com/johnsonlab-ic/singlecell-MR |

For manuscripts utilizing custom algorithms or software that are central to the research but not yet described in published literature, software must be made available to editors and reviewers. We strongly encourage code deposition in a community repository (e.g. GitHub). See the Nature Portfolio guidelines for submitting code & software for further information.

## Data

Policy information about availability of data

All manuscripts must include a data availability statement. This statement should provide the following information, where applicable:

- Accession codes, unique identifiers, or web links for publicly available datasets
- A description of any restrictions on data availability
- For clinical datasets or third party data, please ensure that the statement adheres to our policy

Raw snRNA-seq and genotype data from the Bryois_192 dataset is available as per their publication at the European Genome-Phenome Archive (EGA) under accession code EGAS00001006345 [7]. Raw snRNA-seq and genotype data from the MATTHEWS dataset is hosted at Synapse under accession code syn54083444. Newly generated raw snRNA-seq and associated genotype data (MRC_60 and Roche_PD) is available under accession code EGAS50000000687. Genotype data is considered personal data and is therefore under protected access by the host repository (EGA), where access is subject to the submission of an application delineating the scope of the project and the data required (full details on the portal). Applications are aimed to be reviewed within two weeks.

Processed single-cell expression counts for each dataset and the full set of eQTL summary statistics for both the full and control-only datasets are available at https://zenodo.org/records/13343729 . The full set of published GWAS summary statistics used for the colocalisation and MR analysis as well as links to the original publications are described in Supplementary Table 3.

## Research involving human participants, their data, or biological material

Policy information about studies with human participants or human data. See also policy information about sex, gender (identity/presentation), and sexual orientation and race, ethnicity and racism.

| | |
|---|---|
| Reporting on sex and gender | Yes, sex is included as clinical covariates for the eQTL mapping. |
| Reporting on race, ethnicity, or other socially relevant groupings | Yes, there is a description of genetic ancestry (white european). |
| Population characteristics | Yes, clinical covariates have been included and contain age, sex, genotypic information, disease diagnosis as assessed by neuropathology. All samples were collected post-mortem. |
| Recruitment | This research was conduceted under the oversight of Imperial College Research ethics. |
| Ethics oversight | Imperial College Research Ethics reference: ICREC_14_2_11 |

Note that full information on the approval of the study protocol must also be provided in the manuscript.

# Field-specific reporting

Please select the one below that is the best fit for your research. If you are not sure, read the appropriate sections before making your selection.

☒ Life sciences ☐ Behavioural & social sciences ☐ Ecological, evolutionary & environmental sciences

For a reference copy of the document with all sections, see nature.com/documents/nr-reporting-summary-flat.pdf

# Life sciences study design

All studies must disclose on these points even when the disclosure is negative.

| | |
|---|---|
| Sample size | We performed snRNA-seq on all brain samples available to us, yielding N=409 individuals (391 post quality control). It is the largest dataset to date with almost equal sizes of controls (N = 183) and disease cases (N = 208), allowing to isolate disease-specific effects of feQTLs at cell-type specific level. |
| Data exclusions | Nuclei with less than 500 UMIs in 300 features, and more than 5% Mitochondrial content were excluded. Related individuals based on genotypic data were excluded, and individuals with less than 10 nuclei for a single cell-type were removed. |
| Replication | Replication was made by comparing eQTL discovery to a large-scale eQTL study performed in bulk brain tissue (N = 6,523). Between 72.9-88.7% of cell-type eQTLs (depending on cell type) replicated at FDR < 5%, of which 90.0–98.3 had the same direction of effect. |
| Randomization | Grouping was done based on diagnosis, determined by neuropathology. eQTL discovery was conducted on the full dataset (N = 391) and on the controls-only dataset (N = 183). No other grouping or selection was made. |
| Blinding | Blinding was not implemented to group allocation. However, in our case, the analysis focused on objective genetic and expression data, where researcher bias is unlikely to influence the outcome. Our study design required knowledge of group allocation to conduct separate analyses for controls and the full cohort, which is standard in genetic studies aiming to capture eQTLs across different biological conditions. |

# Reporting for specific materials, systems and methods

We require information from authors about some types of materials, experimental systems and methods used in many studies. Here, indicate whether each material, system or method listed is relevant to your study. If you are not sure if a list item applies to your research, read the appropriate section before selecting a response.

## Materials & experimental systems

| n/a | Involved in the study |
| --- | --- |
| ☒ | ☐ Antibodies |
| ☒ | ☐ Eukaryotic cell lines |
| ☒ | ☐ Palaeontology and archaeology |
| ☒ | ☐ Animals and other organisms |
| ☒ | ☐ Clinical data |
| ☒ | ☐ Dual use research of concern |
| ☒ | ☐ Plants |

## Methods

| n/a | Involved in the study |
| --- | --- |
| ☒ | ☐ ChIP-seq |
| ☒ | ☐ Flow cytometry |
| ☒ | ☐ MRI-based neuroimaging |

## Plants

| | |
| --- | --- |
| Seed stocks | *Report on the source of all seed stocks or other plant material used. If applicable, state the seed stock centre and catalogue number. If plant specimens were collected from the field, describe the collection location, date and sampling procedures.* |
| Novel plant genotypes | *Describe the methods by which all novel plant genotypes were produced. This includes those generated by transgenic approaches, gene editing, chemical/radiation-based mutagenesis and hybridization. For transgenic lines, describe the transformation method, the number of independent lines analyzed and the generation upon which experiments were performed. For gene-edited lines, describe the editor used, the endogenous sequence targeted for editing, the targeting guide RNA sequence (if applicable) and how the editor was applied.* |
| Authentication | *Describe any authentication procedures for each seed stock used or novel genotype generated. Describe any experiments used to assess the effect of a mutation and, where applicable, how potential secondary effects (e.g. second site T-DNA insertions, mosiacism, off-target gene editing) were examined.* |
